# Supplementary material for: Analysis of PPARγ Signaling Activity in Psoriasis
Source: Int J Mol Sci. 2021 Aug 10;22(16):8603. doi: 10.3390/ijms22168603 (PMC8395241; doi:10.3390/ijms22168603)
Supplement: Supplementary file 1 [file ijms-22-08603-s001.zip › Supplemental materials_Analysis of PPARg signaling activity in psoriasis/Pathway models/Models images and html files/Differentiation of psoriatic T cells/9563.html]

PTPN6


# Protein PTPN6

|  |  |
| --- | --- |
| URN | urn:agi-llid:5777 |
| Total Entities | 0 |
| Connectivity | 2384 |
| Name | PTPN6 |
| Description | protein tyrosine phosphatase, non-receptor type 6 |
| Notes | The protein encoded by this gene is a member of the protein tyrosine phosphatase (PTP) family. PTPs are known to be signaling molecules that regulate a variety of cellular processes including cell growth, differentiation, mitotic cycle, and oncogenic transformation. N-terminal part of this PTP contains two tandem Src homolog (SH2) domains, which act as protein phospho-tyrosine binding domains, and mediate the interaction of this PTP with its substrates. This PTP is expressed primarily in hematopoietic cells, and functions as an important regulator of multiple signaling pathways in hematopoietic cells. This PTP has been shown to interact with, and dephosphorylate a wide spectrum of phospho-proteins involved in hematopoietic cell signaling. Multiple alternatively spliced variants of this gene, which encode distinct isoforms, have been reported. [provided by RefSeq, Jul 2008] |
| Primary Cell Localization | Cytoplasm |
| Class | Protein phosphatase |

---

|  |  |
| --- | --- |
| Pathway | DCIR1 (CLEC4A) Signaling |
|  | T-Cell Dependent B-Cell Activation |
|  | Natural Killer Cell Inhibitory Receptor Signaling |
|  | Peripheral T-Cell Tolerance |
|  | Treg-Cell Differentiation |
|  | T-Cell Receptor Signaling |
|  | Toll-like Receptors in Sterile Inflammation |
|  | Do not Eat me\xc2\x9d Signal: Viable Cells Inhibits Phagocytosis |
|  | Hemapoietic Stem Cell -> Lymphoid Multipotent Progenitor Surface Expression Markers |
|  | Atopic Dermatitis Onset |
|  | T-Cells Differentiation Block in Psoriasis |
|  | Proteins with Altered Expression in Psoriasis |
|  | Th0-Cell Aberrant Activation in Systemic Lupus Erythematosus |
|  | Acute Cytotoxic CD8+T-Cell Response against Melanocytes |
|  | T-Cell Cytotoxic Response against Melanocytes in Vitiligo |
|  | Humoral Immunity in Vitiligo |
|  | Treg-Cell Function in Diabetes Mellitus Type 1 |
|  | Natural Killer T-Cell Roles in Diabetes Mellitus Type 1 |
|  | non-Suppressive Treg-Cell in Diabetes Mellitus Type 1 |
|  | TLR2 Signaling in Treg-Cell in Type 1 Diabetes (Animal Model) |
|  | Natural Killer Cell in Diabetes Mellitus Type 1 |
|  | Treg-Cell Activation in Diabetes Mellitus |
|  | Tubular Cell Dysfunction in Progressive Diabetic Nephropathy |
|  | Immune Sustem Activation in Hashimoto's Thyroiditis |
|  | Hashimoto's Thyroiditis |
|  | Immune System Activation in Hyperthyroidism |
|  | CD4+T-Cell Response in Celiac Disease |
|  | Celiac Disease |
|  | Dendritic Cell Function in Ulcerative Colitis |
|  | T-Cell Cytotoxic Response in Ulcerative Colitis |
|  | Follicular Lymphoma |
|  | B-Cells Function in Systemic Scleroderma |
|  | IL6 Promotes Inflammation in Muscular Dystrophies |
|  | CD47 Repression by miRNAs |
|  | Alveolar Macrophage Negative Regulation Declined in COPD |
|  | B-Cell Receptor -> NFATC Signaling |
|  | B-Cell Receptor -> NF-kB Signaling |
|  | CD22 -> STAT Expression Targets |
|  | CD22 -> NF-kB Expression Targets |
|  | CD8 -> AP-1 Expression Targets |
|  | CD72 -> CREB/CREBBP Expression Targets |
|  | CD72 -> NF-kB Expression Targets |
|  | CD72 -> NFATC Expression Targets |
|  | CD72 -> STAT Expression Target |
|  | CD80 -> STAT Expression Targets |
|  | CD81 Expression Targets |
|  | CD80 -> NF-kB Expression Targets |
|  | CD80 -> NFATC Expression Targets |
|  | CD80 -> ATF/CREB/CREBBP Expression Targets |
|  | CD86 -> NFATC Expression Targets |
|  | CD86 -> ATF/CREB/CREBBP Expression Targets |
|  | CD86 -> STAT Expression Targets |
|  | CD86-> NFATC Expression Targets |
|  | CD86 -> NF-kB Expression Targets |
|  | Effector T-cell Inactivation in Cancer Immune Escape |
|  | PTPRJ Expression Targets |
|  | PDCD1 -> ATF/CREB/CREBBP Expression Targets |
|  | PDCD1 -> STAT Expression Targets |
|  | PDCD1 -> NF-kB Expression Targets |
|  | PDCD1 -> NFATC Expression Targets |
|  | PDCD1 -> AP-1 Expression Targets |
|  | T-Cell Receptor -> AP-1 Signaling |
|  | T-Cell Receptor -> NF-kB Signaling |
|  | T-Cell Receptor -> NFATC Signaling |
|  | T-Cell Receptor -> STAT Signaling |
|  | T-Cell Receptor -> ATF/CREB Signaling |
|  | T-Cell Receptor -> CREBBP Signaling |
|  | ROS in Angiotensin Mediated Cardiovascular Remodeling and Hypertrophy |
|  | ace2 |
|  | sialic |
|  | PPAR Psoriasis |
|  | New Pathway (3) |
|  | 1\_Differentiation of psoriatic T cells |
|  | 1\_The onset of atopic dermatitis |
|  | 1\_Th2 cell response in asthma |
|  | 2\_Intestine epithelial cells drive immunological responses in ulcerative colitis |
|  | 1\_CD4+ T cell and epithelial cell response in celiac disease |
|  | 2\_Defective tolerance of autoreactive T-cell in T1D |
|  | Differentiation of psoriatic T cells |

---

|  |  |
| --- | --- |
| Group | Genes Frequently Hypermethylated in Follicular Lymphoma |

---

|  |  |
| --- | --- |
| MedScan ID | 5777 |

---

|  |  |
| --- | --- |
| LocusLink ID | 5777 |
|  | 15170 |
|  | 116689 |

---

|  |  |
| --- | --- |
| Alias | SH2-containing tyrosine phosphatase SHP-1 |
|  | 70Z-SHP |
|  | protein tyrosine phosphatase, non receptor type 6 |
|  | SH-PTP1 |
|  | Src homology region 2 domain (SH2)-containing tyrosine phosphatase-1 |
|  | hematopoietic phosphotyrosine phosphatase SHP-1 |
|  | hematopoietic cell protein-tyrosine phosphatase |
|  | SHP-1Ls |
|  | MGC124580 |
|  | PTP-1Cs |
|  | Src homology domain 2 (SH2)-containing tyrosine phosphatase-1 |
|  | PTPN6 |
|  | Src homology region 2 domain-containing tyrosine phosphatase SHP-1 |
|  | Src homology domain 2 containing protein phosphatase 1 |
|  | motheaten |
|  | PTPTY-42 |
|  | HCPH |
|  | SH2 phosphatase 1 |
|  | Src homology region domain 2 (SH2)-containing tyrosine phosphatase-1 |
|  | hemopoietic cell phosphatase |
|  | Src homology domain 2-containing tyrosine phosphatase SHP-1 |
|  | SHP I |
|  | Src homology region domain 2-containing tyrosine phosphatase SHP-1 |
|  | protein tyrosine phosphatase, nonreceptor type 6 |
|  | SHP1 protein tyrosine phosphatase |
|  | Src homology region domain 2 containing protein phosphatase 1 |
|  | phosphatase SHP1 |
|  | SHP-1L |
|  | protein-tyrosine phosphatase 1C |
|  | Ptph6 |
|  | HPTP1C |
|  | protein tyrosine phosphatase, non receptor type VI |
|  | protein-tyrosine phosphatase SHP-1 |
|  | Src homology region 2 domain-containing protein phosphatase 1 |
|  | SHP1 |
|  | SHP 1 phosphatase |
|  | SH2 phosphatase I |
|  | protein tyrosine phosphatase, nonreceptor type VI |
|  | Ptp1C |
|  | hematopoietic cell phosphatase |
|  | 70 kda SHP-1L protein |
|  | hematopoietic cell phophatase |
|  | SH-PTP I |
|  | HCP |
|  | SHP-1 |
|  | PTP-1C |
|  | tyrosine-protein phosphatase non-receptor type 6 |
|  | me |
|  | protein tyrosine phosphatase, non-receptor type 6 |
|  | protein tyrosine phosphatase, non-receptor type VI |
|  | motheaten protein |

---

|  |  |
| --- | --- |
| EC Number | 3.1.3.48 |

---

|  |  |
| --- | --- |
| GO ID | 0042169 |
|  | 0017124 |
|  | 0050839 |
|  | 0140031 |
|  | 0001784 |
|  | 0019901 |
|  | 0004725 |
|  | 0005001 |
|  | 0050853 |
|  | 0007186 |
|  | 0031295 |
|  | 0033277 |
|  | 0006915 |
|  | 0030154 |
|  | 0008283 |
|  | 0071345 |
|  | 0019221 |
|  | 0002244 |
|  | 0035556 |
|  | 0050900 |
|  | 0035855 |
|  | 0042267 |
|  | 0043407 |
|  | 0042130 |
|  | 0050860 |
|  | 0008285 |
|  | 0002924 |
|  | 0032715 |
|  | 0050732 |
|  | 0032720 |
|  | 0043312 |
|  | 0035335 |
|  | 0018108 |
|  | 0030168 |
|  | 0070527 |
|  | 0030220 |
|  | 0033630 |
|  | 0008284 |
|  | 0014068 |
|  | 0006470 |
|  | 0045577 |
|  | 0070372 |
|  | 2000045 |
|  | 0051279 |
|  | 0060338 |
|  | 0042105 |
|  | 0005911 |
|  | 0005737 |
|  | 0005829 |
|  | 0070062 |
|  | 0005576 |
|  | 0016020 |
|  | 0005730 |
|  | 0005654 |
|  | 0005634 |
|  | 0032991 |
|  | 0035580 |
|  | 1904724 |
|  | 0016787 |
|  | 0046703 |
|  | 0016791 |
|  | 0004721 |
|  | 0016311 |
|  | 0043409 |
|  | 0050859 |
|  | 0009611 |
|  | 0060397 |
|  | 0007596 |
|  | 0060333 |
|  | 0060334 |
|  | 0060337 |
|  | 0007165 |
|  | 0005622 |
|  | 0034329 |
|  | 0004872 |

---

|  |  |
| --- | --- |
| KEGG ID | rno:116689 |
|  | hsa:5777 |
|  | mmu:15170 |

---

|  |  |
| --- | --- |
| Organism | Homo sapiens {Organism urn:agi-taxid:9606} |
|  | Mus musculus {Organism urn:agi-taxid:10090} |
|  | Rattus norvegicus {Organism urn:agi-taxid:10116} |
|  | Homo sapiens |
|  | Mus musculus |
|  | Rattus norvegicus |

---

|  |  |
| --- | --- |
| Mouse chromosome position | 6 59.17 cM |
|  | 6 60.22 cM |

---

|  |  |
| --- | --- |
| OMIM ID | 176883 |

---

|  |  |
| --- | --- |
| Rat chromosome position | 4q42 |

---

|  |  |
| --- | --- |
| Hugo ID | 9658 |
|  | HGNC:9658 |

---

|  |  |
| --- | --- |
| Human chromosome position | 12p13.31 |
|  | 12p13 |

---

|  |  |
| --- | --- |
| Swiss-Prot Accession | P29350 |
|  | Q53XS4 |
|  | P29350.1 |
|  | P29351 |
|  | Q3UB72 |
|  | Q3UCJ0 |
|  | P29351.2 |
|  | P81718 |
|  | P81718.1 |
|  | A8K306 |
|  | G3V0F8 |
|  | Q969V8 |
|  | Q9UK67 |
|  | O35128 |
|  | Q63872 |
|  | Q63873 |
|  | Q63874 |
|  | Q921G3 |
|  | Q9QVA6 |
|  | Q9QVA7 |
|  | Q9QVA8 |
|  | Q9R0V6 |
|  | Q53EL0 |
|  | Q3TE70 |
|  | Q499N7 |
|  | Q8R5H0 |

---

|  |  |
| --- | --- |
| PIR ID | B42031 |
|  | A44390 |

---

|  |  |
| --- | --- |
| GenBank ID | NC\_000012 |
|  | NM\_080548 |
|  | NP\_536858 |
|  | XM\_011520988 |
|  | XP\_011519290 |
|  | NM\_080549 |
|  | NP\_536859 |
|  | XM\_006718994 |
|  | XP\_006719057 |
|  | NM\_002831 |
|  | NP\_002822 |
|  | XM\_024449106 |
|  | XP\_024304874 |
|  | AB019535 |
|  | AB079851 |
|  | BAC81774 |
|  | BAC81775 |
|  | AH003242 |
|  | AAA82879 |
|  | CH471116 |
|  | EAW88700 |
|  | EAW88701 |
|  | EAW88702 |
|  | EAW88703 |
|  | EAW88704 |
|  | HI573994 |
|  | CBX47486 |
|  | HI573996 |
|  | CBX47487 |
|  | U47924 |
|  | AAB51322 |
|  | AAB51323 |
|  | X82818 |
|  | AF178946 |
|  | AAD53317 |
|  | AK223629 |
|  | BAD97349 |
|  | AK290421 |
|  | BAF83110 |
|  | AK298470 |
|  | BAG60682 |
|  | AK300167 |
|  | BAG61949 |
|  | AK308230 |
|  | AK309037 |
|  | AK313379 |
|  | BAG36177 |
|  | BC002523 |
|  | AAH02523 |
|  | BC007667 |
|  | AAH07667 |
|  | BG754792 |
|  | BM006699 |
|  | BM742181 |
|  | BT007390 |
|  | AAP36054 |
|  | CR986427 |
|  | DA431502 |
|  | DA868001 |
|  | DC297194 |
|  | M74903 |
|  | AAA35963 |
|  | M77273 |
|  | AAA36610 |
|  | P29350 |
|  | NC\_000072 |
|  | NM\_001077705 |
|  | NP\_001071173 |
|  | NM\_013545 |
|  | NP\_038573 |
|  | AC002397 |
|  | AAC36008 |
|  | AAC36009 |
|  | AC164157 |
|  | AH007124 |
|  | AAD00151 |
|  | AAD00152 |
|  | CH466523 |
|  | EDK99751 |
|  | AK132509 |
|  | BAE21210 |
|  | AK150509 |
|  | BAE29622 |
|  | AK151077 |
|  | BAE30092 |
|  | AK151623 |
|  | AK159686 |
|  | AK159833 |
|  | AK167923 |
|  | AK169804 |
|  | BAE41378 |
|  | BC012660 |
|  | AAH12660 |
|  | BY035180 |
|  | BY747031 |
|  | M68902 |
|  | AAA37796 |
|  | M90389 |
|  | AAA40007 |
|  | S63763 |
|  | AAP13944 |
|  | S63764 |
|  | AAP13945 |
|  | S63803 |
|  | AAP13953 |
|  | P29351 |
|  | NC\_005103 |
|  | NM\_053908 |
|  | NP\_446360 |
|  | AC\_000072 |
|  | AAHX01032846 |
|  | AAHX01032847 |
|  | AC129138 |
|  | CH473964 |
|  | EDM01944 |
|  | EDM01945 |
|  | AF468653 |
|  | AAL77056 |
|  | BC099824 |
|  | AAH99824 |
|  | FQ232747 |
|  | U77038 |
|  | AAD00262 |
|  | P81718 |
|  | XR\_931520 |
|  | XM\_011520989 |
|  | XP\_011519291 |
|  | NC\_018923 |
|  | AMYH02025958 |
|  | U15528 |
|  | AAA82880 |
|  | U15529 |
|  | U15530 |
|  | U15531 |
|  | U15532 |
|  | U15533 |
|  | U15534 |
|  | U15535 |
|  | U15536 |
|  | U15537 |
|  | DQ891946 |
|  | ABM82872 |
|  | DQ895133 |
|  | ABM86059 |
|  | AC\_000028 |
|  | AAHY01057229 |
|  | U65951 |
|  | U65952 |
|  | U65953 |
|  | U65954 |
|  | U65955 |
|  | AC\_000144 |
|  | ABBA01017669 |
|  | ABBA01017670 |
|  | NW\_003871083 |
|  | NT\_009759 |
|  | NW\_925295 |
|  | NW\_001838050 |
|  | AC\_000055 |
|  | CR590933 |
|  | Q53EL0 |
|  | Q53XS4 |
|  | Q9UK67 |
|  | NT\_039353 |
|  | NW\_001030811 |
|  | Q3TE70 |
|  | Q3UB72 |
|  | Q3UCJ0 |
|  | NW\_047696 |
|  | NW\_001084832 |
|  | Q499N7 |
|  | Q8R5H0 |

---

|  |  |
| --- | --- |
| Swiss-Prot ID | PTN6\_RAT |
|  | PTN6\_HUMAN |
|  | PTN6\_MOUSE |
|  | Q3UCJ0\_MOUSE |
|  | Q53XS4\_HUMAN |
|  | Q9UK67\_HUMAN |
|  | A8K306\_HUMAN |

---

|  |  |
| --- | --- |
| Cell Localization | Cytoplasm |
|  | Nucleus |

---

|  |  |
| --- | --- |
| Ensembl ID | ENSG00000111679 |
|  | ENSP00000326010.9 |
|  | ENST00000318974.14 |
|  | ENSP00000382376.1 |
|  | ENST00000399448.5 |
|  | ENSP00000391592.1 |
|  | ENST00000456013.5 |
|  | ENSMUSG00000004266 |
|  | ENSMUSP00000129124.2 |
|  | ENSMUST00000171549.8 |
|  | ENSMUSP00000108103.3 |
|  | ENSMUST00000112484.9 |
|  | ENSRNOG00000014294 |
|  | ENSRNOP00000059867.1 |
|  | ENSRNOT00000065416.3 |
|  | ENST00000318974.13 |
|  | ENSP00000326010 |
|  | ENST00000318974 |
|  | ENSP00000382376 |
|  | ENST00000399448 |
|  | ENSP00000391592 |
|  | ENST00000456013 |
|  | ENSMUSP00000129124 |
|  | ENSMUST00000171549 |
|  | ENSMUSP00000108103 |
|  | ENSMUST00000112484 |
|  | ENSRNOP00000059867 |
|  | ENSRNOT00000065416 |

---

|  |  |
| --- | --- |
| MGI ID | MGI:96055 |
|  | 96055 |

---

|  |  |
| --- | --- |
| RGD ID | 620660 |

---

|  |  |
| --- | --- |
| Unigene ID | Hs.63489 |
|  | Rn.18985 |
|  | Mm.271799 |

---

|  |  |
| --- | --- |
| Homologene ID | 56589 |

---

|  |  |
| --- | --- |
| Shape | 2Triangles |

---

|  |  |
| --- | --- |
| IPI ID | IPI00225419 |
|  | IPI00225422 |
|  | IPI00310909 |
|  | IPI00183046 |
|  | IPI00218604 |
|  | IPI00396552 |
|  | IPI00941721 |
|  | IPI00339114 |
|  | IPI00795221 |
|  | IPI00789746 |

---
